# A novel preclinical model of the normal human breast

**DOI:** 10.1007/s10911-024-09562-4

**Published:** 2024-05-02

**Authors:** Anthony J. Wilby, Sara Cabral, Nastaran Zoghi, Sacha J. Howell, Gillian Farnie, Hannah Harrison

**Affiliations:** 1 Division of Cancer Sciences, Manchester Cancer Research Centre, University of Manchester, Oglesby Cancer Research Building, Wilmslow Road, Manchester, M20 4GJ United Kingdom; 2 Manchester Breast Centre, University of Manchester, Wilmslow Road, Manchester, M20 4GJ United Kingdom; 3 Henry Royce Institute, University of Manchester, Oxford Road, Manchester, M13 9PL United Kingdom; 4 Department of Materials & Institute of Biotechnology, University of Manchester, Manchester, M1 7DN United Kingdom; 5 NIHR Manchester Biomedical Research Centre, Manchester Academic Health Science Centre, Central Manchester University Hospitals NHS Foundation Trust, 29 Grafton St, Manchester, M13 9WU United Kingdom; 6 The Nightingale and Prevent Breast Cancer Centre, Manchester University NHS Foundation Trust, Manchester, M23 9LT United Kingdom; 7 Cancer Research Horizons, The Francis Crick Institute, 1 Midland Road, Manchester, NW1 1AT United Kingdom

**Keywords:** Normal breast, Explants, Prevention, Risk-reduction, In vitro modelling

## Abstract

Improved screening and treatment have decreased breast cancer mortality, although incidence continues to rise. Women at increased risk of breast cancer can be offered risk reducing treatments, such as tamoxifen, but this has not been shown to reduce breast cancer mortality. New, more efficacious, risk-reducing agents are needed. The identification of novel candidates for prevention is hampered by a lack of good preclinical models. Current patient derived in vitro and in vivo models cannot fully recapitulate the complexities of the human tissue, lacking human extracellular matrix, stroma, and immune cells, all of which are known to influence therapy response. Here we describe a normal breast explant model utilising a tuneable hydrogel which maintains epithelial proliferation, hormone receptor expression, and residency of T cells and macrophages over 7 days. Unlike other organotypic tissue cultures which are often limited by hyper-proliferation, loss of hormone signalling, and short treatment windows (< 48h), our model shows that tissue remains viable over 7 days with none of these early changes. This offers a powerful and unique opportunity to model the normal breast and study changes in response to various risk factors, such as breast density and hormone exposure. Further validation of the model, using samples from patients undergoing preventive therapies, will hopefully confirm this to be a valuable tool, allowing us to test novel agents for breast cancer risk reduction preclinically.

**Supplementary Information:**

The online version contains supplementary material available at 10.1007/s10911-024-09562-4.

## Introduction

Over the last 40 years, improved screening and treatment have significantly decreased breast cancer mortality in the UK [1, 2] with a combined 41% decrease since the 1970s in both females and males[3]. Despite this, the incidence of breast cancer continues to rise [4, 5] with an 18% increase in the UK between 1993 and 2016 [6]. This increase in incidence can, in part, be attributed to improved screening techniques, however, it highlights the importance of prevention and risk-reduction interventions. Women at high risk of breast cancer can be offered risk-reducing agents including selective oestrogen receptor modulators, tamoxifen and raloxifene, and an aromatase inhibitor, anastrozole [7]. While they help reduce the risk of primary breast cancer by 30–50%, they have not been shown to decrease mortality [8, 9]. New preventative agents that reduce the risk of potentially fatal breast cancers are required [10]. Such agents require extensive preclinical testing before they can be used in the clinic. Current in vitro and in vivo models of the normal human breast do not fully recapitulate the human breast extracellular matrix (ECM) and its complex cellular environment. In the cancer treatment setting, this is thought to contribute to a poor rate of translation from preclinical studies to human trials with around 90–95% of drugs failing before reaching the clinic [11]. To discover new preventative agents capable of successful translation to the clinic, a model which overcomes these limitations is required.

Normal breast tissues xenografted into immunocompromised mice allow for human epithelial duct persistence, but the ECM and stromal cells are murine and lack most host immune cells [12, 13]. Each of these components is required for normal tissue homeostasis and plays a role in cancer development and progression [14–17]. Their absence may result in differential responses to therapies compared with the intact human gland in vivo.

In vitro models, such as patient-derived organoids, use tissue which is enzymatically digested prior to culture, where it is grown in rodent-derived ECM supports, such as Matrigel. This Setup lacks many of the normal human ECM components and cell–cell interactions. Organotypic tissue slice and explant models retain the complexity of the normal breast whilst supporting tissue on gelatin sponges. They are predominantly used for cancer research, but bring their own challenges, such as abnormal proliferation, loss of hormone signalling and loss of viability after 96h [18] and employ unphysiological levels of glucocorticoids which may interfere with signalling by other steroids.[19].

Tissue stiffness is a key factor in breast carcinogenesis [12, 13, 20] and the maintenance of hormone receptor expression in vitro [21], meaning selecting the correct matrix and the correct elastic modulus is of utmost importance.

Intact breast organoid culture has delivered an improved in vitro model for hormone investigations but there are few models that accurately recapitulate the structure of the ECM. These models also have a time-limited treatment window of between 24 and 72h. Additionally, whilst models have been produced that incorporate other cell types, for example stromal cells [22] and fibroblasts [23], no current model accurately reproduces the entire repertoire of cell types, limiting our ability to model normal breast physiology [24–26].

We describe here a tissue explant model utilising a tuneable hydrogel which preserves cellular heterogeneity and hormone signalling for 7 days. This model will be used to identify novel agents to translate into the clinical prevention setting and to study how risk factors, such as breast density and exposure to hormones or chemicals impact on cancer development.

## Materials and methods

### Explant culture

**Fig. 1:**
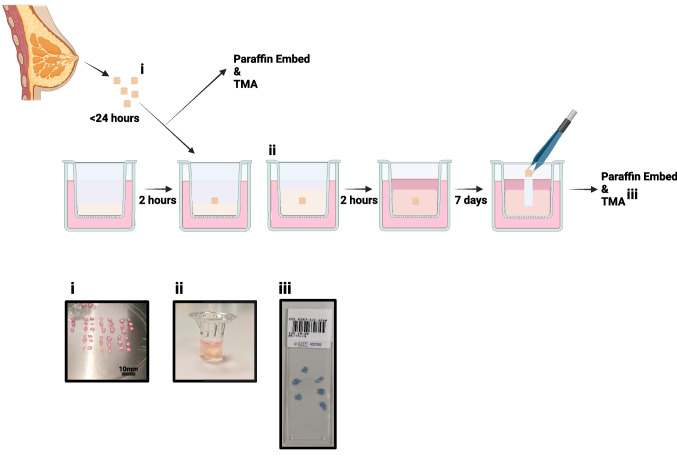
Explant model schematic: Individual steps are highlighted from tissue collection to fixing for immunohistochemistry. **i**. shows example of tissue processing, **ii**. tissue can be seen within the Boyden chamber, encased in hydrogel and **iii**. shows an example of the tissue microarray (TMA) produced for each sample

Figure 1 shows the culture procedure we have developed.

### Tissue Collection and Dissection

Normal non-cancerous breast tissue was collected following risk-reduction surgery. Research samples were obtained from the Manchester Cancer Research Centre (MCRC) Biobank with fully informed consent. Ethical approval for the study was granted by the MCRC Biobank under authorisation number 18/NW/0092. Sample details are displayed in Table 1.

**Table 1:** Patient samples. TMA – Tissue microarray, BC – breast cancer, NR—not recorded

Sample Identification Number	TMA Number	Age	Children	Risk
BB7108T1N	108	26	0	Family History
BB7110T1N	110	33	0	BRCA1
BB7115T1N	115	36	2	BRCA2
BB7125T1N	125	30	NR	BRCA2
BB7130T1N	130	28	0	BRCA1
BB7134T1N	134	46	NR	Previous contralateral BC
BB7138T1N	138	42	NR	BRCA1
BB7156T1N	156	37	2	p53
BB7167T1N	167	28	2	BRCA2
BB7171T1N	171	38	1	BRCA2
BB7181T1N	181	40	NR	PALB2
BB7188T1N	188	34	1	Family History
BB7196T1N	196	35	NR	BRCA2

Tissue was placed immediately into collection medium consisting of DMEM High Glucose (SIGMA, D6546) with 100U/mL Penicillin/100 µg/mL Streptomycin (SIGMA, P0781) and stored for up to 24 h at 4°C. Excess adipose tissue was removed, and tissue was cut into 2–4 mm^3^ pieces before culture.

### 3D Matrix

An animal-free hydrogel (VitroGel RGD, TebuBio, TWG003) was used to provide support to explants. The hydrogel was mixed with 0.5 × PBS to achieve the desired elastic modulus (which is representative of stiffness), according to the manufacturer’s instructions [27]. This was then mixed with medium to initiate hydrogel gelation and 100 µL was immediately pipetted into Boyden chambers suspended over a well containing 700 µL of medium. Chambers were incubated for 2 h at 37°C to allow the hydrogels to set. The same procedure was followed to overlay 150 µL of hydrogel on top of the explant and, once the hydrogel was set, 200 µL of medium was added to the top. During culture, 50% of the medium below and above the explant was refreshed every 2–3 days.

### Culture Media

Explant medium (ExM): DMEM/F12 (Thermo, 11330032) containing B27 supplement (no vitamin A; Invitrogen, Paisley, UK, 12587010), 2 mM L-glutamine (SIGMA, G7513) and 100 U/mL Penicillin/100 µg/mL Streptomycin (SIGMA, P0781).

Clevers’ medium (CM[28]): DMEM/F12 containing 5% R-spondin conditioned medium, 5 nM neuregulin (Peprotech, 100–03), 5 ng/mL epidermal growth factor (Peprotech, AF-100–15), 100 ng/mL noggin (Peprotech, 120-10C), 500 nM A83-01 (Tocris, 2939), 5 µM Y27632 (Abmole, S1049), 500 nM SB202190 (Sigma, S7067), 1 × B27 (with vitamin A, Gibco, 1750444), 1.25 nM N-acetylcysteine (Sigma, A9165), 5 mM nicotinamide (Sigma, N0636), 1 × Glutamax (Invitrogen, 12634–034), 10 mM HEPES (Invitrogen, 15,630–056), 100 U/mL Penicillin/100 µg/mL Streptomycin and 50 ng/mL FGF2 (Thermo, 100-18B).

FCS medium: DMEM/F12 and 10% foetal calf serum (FCS, Thermo, 10270106), 100 U/mL Penicillin/100 µg/mL Streptomycin.

### Hormone Responsiveness assays

For activation and inhibition studies, 10 nM 17β-oestradiol (SIGMA, E2758) and/or 100 nM fulvestrant (SIGMA, I4409) was added to the medium following explant encapsulation in its hydrogel support and was refreshed with each medium change.

### Immunohistochemistry: Staining

Tissue was removed from the hydrogel, formalin-fixed and paraffin-embedded, with 6 explants per block, and 4 µm slices were prepared for immunohistochemistry. Staining was performed using the Bond Max autostainer (Leica) and Ventana Discovery autostainer (Roche). TMAs were scanned using the Olympus VS120.

Staining on the Leica Bond Max was performed with 20 min of antigen retrieval at pH6 (Ki67, progesterone receptor (PR) and cleaved caspase 3) and pH9 (oestrogen receptor α (ERα)). Primary antibodies: mouse α-ERα (6F11, Life Technologies, MA513304) 1:200, mouse α-Ki67 (MIB-1, DAKO, M7240) 1:100, mouse α-PR (636, DAKO, M3569) 1:500, rabbit α-cleaved caspase 3, 1:200 (5A1E, New England Biolabs, 9664S) and EnVision + Single Reagents (HRP, Mouse, Agilent, K400111-2) used as secondary, following the manufacturer’s instructions.

Staining on the Ventana was performed for CD4 and CD8 using an *ultra*View Universal DAB Detection Kit (Roche, 760–500) and CD68 using an *OptiView* DAB IHC Detection Kit (Roche, 76–700). Slides were deparaffinised, antigens were retrieved using standard cell conditioning (CC1), primary antibody incubations were performed for 16 min, and bluing with haematoxylin II was performed for 4 min. The following primary antibodies were used according to the manufacturer's instructions: α-CD4 (SP35, Roche, 790–4423), α-CD8 (SP57, Roche, 790–4460) and α-CD68 (KP-1, Roche, 790–2931).

### Immunohistochemistry: Scoring

Scoring was performed, and percentage positive calculated, in https://imagej.net/ij/ at 10 × magnification. All epithelial cells in the explant (ERα, PR, Ki67, Caspase) were counted and immune cells (CD4, CD8, CD68) were treated as a single population whether inter- or intraductal. Fold change from control was calculated to demonstrate changes following culture.

### Rheology

To test the elastic modulus of the low, moderate and high stiffness gels, 500 µL samples were prepared using ExM in Boyden chamber hanging inserts, as described above. The inserts were incubated at 37°C for 24 h prior to rheological testing. Hydrogels were removed from the hanging insert and transferred to the rheometer. The 25 mm upper parallel plate of the rheometer was lowered to the desired trim gap size of 500 µm, and the gels were allowed to equilibrate for 3 min at room temperature. Single frequency (1Hz) amplitude sweeps were performed between 0.001% and 100% shear strain using an Anton Parr MCR 302E rheometer.

### Statistical Analysis

One-way ANOVA tests were performed, with pairing of samples, and comparisons made to day 0 or untreated sample as appropriate. A Dunnett’s correction for multiple testing was performed. For rheology, measurements were repeated four times, and a one-way ANOVA was performed on the linear viscoelastic regions of the gels. Significance is highlighted in each figure; *P < 0.05, **P < 0.01, ***P < 0.001.

## Results

### Model Development: Culture Medium Selection

We began model development by altering the media within our model, whilst maintaining a consistent hydrogel elastic modulus (Vitrogel RGD:0.5X PBS:medium 1:1:1, described as “moderate” according to manufacturer’s instructions, see Table 2). We compared 3 culture media: explant medium (ExM), Clevers’ medium (CM[28]), which is commonly used for organoid culture and FCS medium, which is commonly used in our lab for 3D Matrigel cell culture.

**Table 2:** Hydrogel make up

Gel	VitroGel RGD (µL)	0.5X PBS (µL)	ExM (µL)	Elastic modulus (Pa)
High	200	0	100	1472.11 ± 26.65
Moderate	100	100	100	413.78 ± 9.73
Low	100	200	100	244.43 ± 6.99

**Fig. 2:**
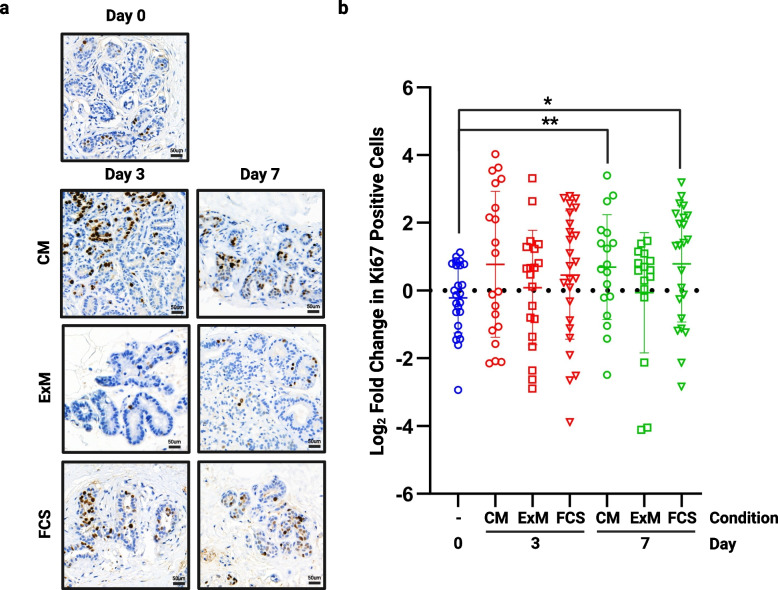
The effect of medium on proliferation. Following 3 and 7 days of culture in each of the media tested, tissue was fixed and assessed for proliferative rate using Ki67. **a**) shows representative images from each medium at each time point (TMA108). **b**) shows fold change in proliferation from day 0 in multiple cores from 4 patient samples. Significant increases in proliferation were seen in both Clevers’ medium (CM) and FCS medium at day 7. *P < 0.05 ** *P* < 0.01. Scale bar shows 50 µm

Proliferation in the different media was assessed by staining tissue for Ki67, and representative images are shown in Fig. 2A. Proliferation was significantly increased in both CM and FCS medium at day 7 whilst in ExM, proliferation was unchanged at both time points (Fig. 2B, n = 4). In the remaining studies reported here, we selected ExM to be our standard explant medium, as this maintained our cultured explant proliferation at a rate similar to that measured in matched non-cultured breast tissue.

### Model Development: 3D Matrix

**Fig. 3:**
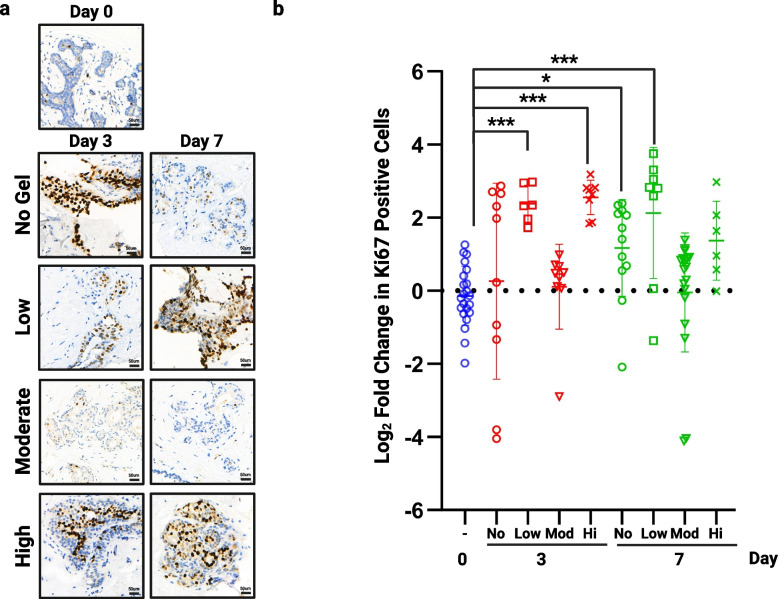
The effect of hydrogel support on proliferation**.** Following 3 and 7 days of culture in no hydrogel or each of the 3 hydrogel densities tested, tissue was fixed and assessed for proliferative rate using Ki67. **a**) shows representative images from each hydrogel support at each time point (TMA110). **b**) shows fold change in proliferation from day 0 in multiple cores from 3 patient samples. Significant increases in proliferation were seen when tissue was cultured with no support at day 7, within low support hydrogel at days 3 and 7 and in high support hydrogels at day 3. No change was seen using our moderate hydrogel (413.78 Pa). *P < 0.05 ** *P* < 0.01 *** *P* < 0.001. Scale bar shows 50 µm

To ensure we selected the best elasticity of hydrogel for our model we tested three preparations, termed low, moderate and high, against tissue cultured in no matrix (Table 2). The moderate support was clearly superior at preventing hyper-proliferation when compared to standard membrane supported tissue (no hydrogel) or the low and high hydrogels, with significant changes in Ki67 expression in these conditions over 7 days (Fig. 3, n = 3). Rheology was performed on each hydrogel mix (Suppl. Figure 1) and showed that the moderate hydrogel had an elastic modulus of 413.78 Pa ± 9.73 and that even small differences can significantly affect proliferation with increased proliferation in low and high gels which measured 244.43 Pa ± 6.99 and 1472.11 Pa ± 26.65 respectively.

### Culture Validation: Proliferation and Viability are Maintained Over 7 days

**Fig. 4:**
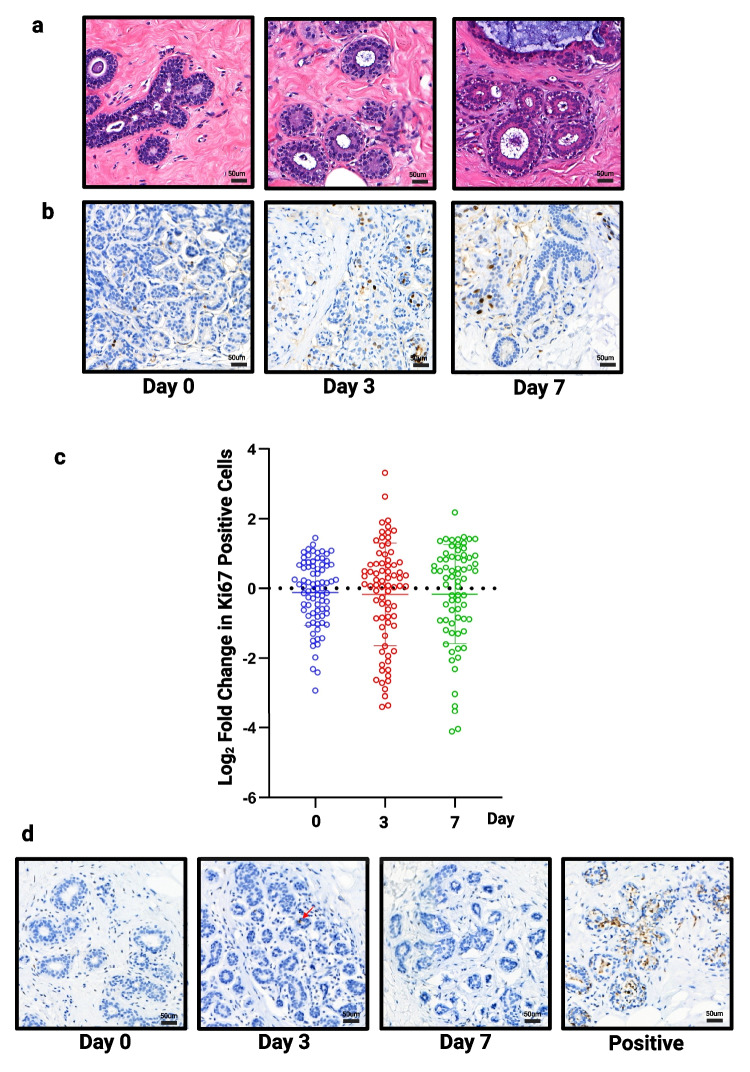
Assessment of structure, proliferation and apoptosis in all samples**. a**) Representative images of H&E staining (TMA134) and **b**) Ki67 staining following 3 and 7 days of culture (TMA134) in optimised conditions. **c**) No significant change in proliferation was seen (*n* = 13). **d**) Representative images of caspase staining following 3 and 7 days of culture (TMA115), red arrow highlights single positive cell. Tissue cultured in FCS medium on day 3 used as an example of positive staining. Scale bar shows 50 µm

To validate our selected culture conditions, using ExM and moderate hydrogel, we first performed staining on our full tissue panel (*n* = 13) with H&E (Fig. 4A) and Ki67 at 0, 3 and 7 days (Fig. 4B). By day 7, there is evidence of vacuolation within the myoepithelial cells, which occurs during the luteal phase in normal tissue [29, 30], and may suggest the tissue is responding to progesterone, a component of B27, in the medium. No significant change in proliferation was observed over 7 days (Fig. 4C).

Cleaved caspase 3 was used to assess cell viability. The number of cells staining positive for caspase was low and zero in many slices, meaning we were unable to perform statistical testing but drew the conclusion that no change was seen in viability over 7 days. Figure 4D shows representative images of explants cultured in our selected conditions as well as an example of positive staining when explants were cultured in FCS medium.

### Culture Validation: Expression of Hormone Receptors is Maintained During Explant Culture

**Fig. 5:**
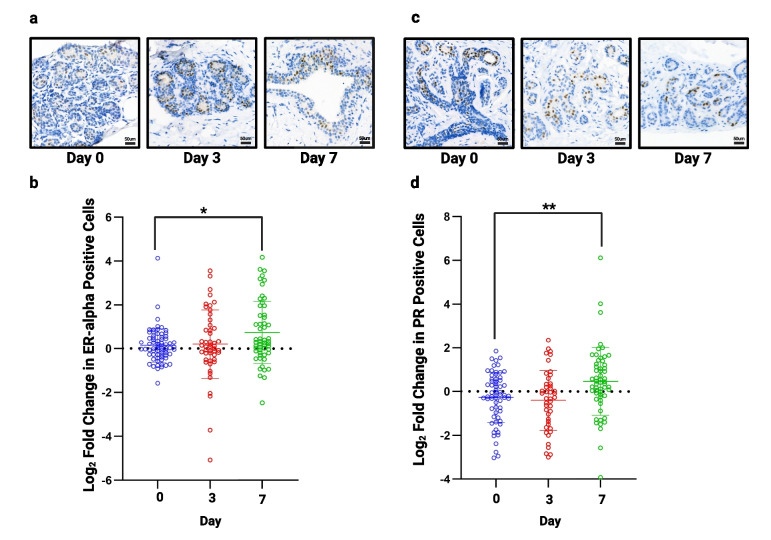
Assessment of hormone receptor expression in all samples. **a**) Representative images of ERα staining following 3 and 7 days of culture (TMA138). **b**) No significant change in ERα was seen at day 3 but there was a small, but significant increase at day 7 (*n* = 13). **c**) Representative images of PR staining following 3 and 7 days of culture (TMA134). **d**) No significant change in progesterone receptor was seen at day 3 but a small but significant, increase is seen at day 7 (*n* = 13). *P < 0.05 ** *P* < 0.01. Scale bar shows 50 µm

Next, we stained our TMAs for ERα and PR to confirm whether their expression remained unchanged during culture. Figure 5 shows representative images of ERα and PR staining. A small, but significant, increase in both ERα (Fig. 5B) and PR (Fig. 5D) was seen after 7 days (*n* = 13), but was unchanged at day 3.

### Culture Validation: Explants Remain Responsive to Oestrogen

To assess whether the tissue explants remained responsive to oestrogen, 10 nM 17β-oestradiol (E2) was added to the cultures for 7 days in the presence or absence of 100 nM fulvestrant, an anti-oestrogen. The proliferative response was assessed by Ki67 staining, and the transcriptional response was assessed by staining for PR, a transcriptional target of ERα.

**Fig. 6:**
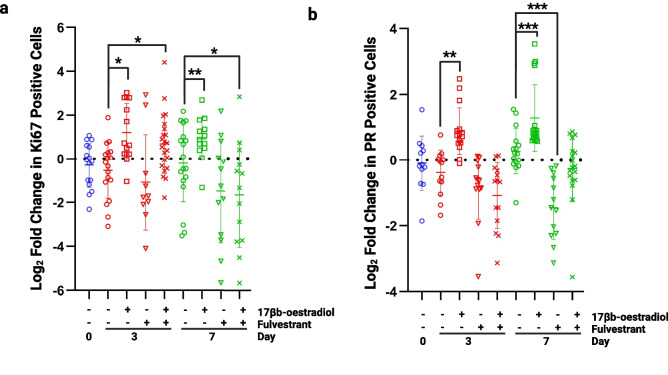
Assessment of oestrogen responsiveness. **a**) Proliferation was significantly increased at days 3 and 7 following the addition of 10 nM 17β-oestradiol. At day 3 proliferation remained significantly increased following the addition of 100 nM fulvestrant but at day 7, proliferation had fallen below control levels (day 7, no 17β-oestradiol). **b**) Progesterone receptor (PR) expression was significantly increased in the presence of 10 nM 17β-oestradiol at days 3 and 7 and this effect was blocked by 100 nM fulvestrant. *P < 0.05 **P < 0.01 ***P < 0.001

Following the addition of E2, proliferation was significantly increased at days 3 and 7 showing a response to hormone stimulation (Fig. 6A). This increase in proliferation was blocked with the addition of fulvestrant at day 7 confirming the effect was a direct influence of E2 acting through ERα. Similar changes were seen when measuring the expression level of PR; the number of positive cells increased significantly with the addition of E2 at day 3 and 7 (Fig. 6B). This increase was lost in the presence of fulvestrant, with expression levels being significantly reduced compared to controls.

### Culture Validation: Immune cells can be seen within tissue explants

**Fig. 7:**
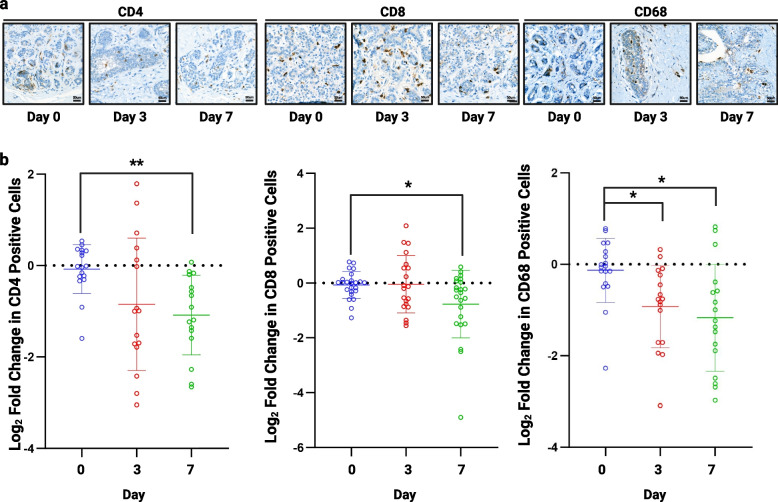
Assessment of immune cell infiltration**. a**) Representative images of CD4, CD8 and CD68 staining following 3 and 7 days of culture (TMA156). **b**) A significant decrease was seen in CD4 (n = 4) and CD8 (*n* = 7) cells after 7 days of culture and CD68 (*n* = 4) at 3 and 7 days. *P < 0.05 **P < 0.01. Scale bar shows 50 µm

The TMAs were stained for recognised immune cell markers including CD4, T helper cells (n = 4), CD8, cytotoxic T cells (n = 7), and CD68, macrophages (n = 4) (Fig. 7A). CD4 and CD8 T cells persisted throughout 7 days of culture but there was a significant decrease in these cells by day 7 (Fig. 7B). Macrophages (CD68) were also seen throughout culture with a small but significant decrease in the numbers seen (Fig. 7B).

## Discussion

We have developed a robust in vitro model of human breast tissue that maintains cell viability and tissue morphology like that of uncultured tissue over 7 days. Typically, proliferation, measured using Ki67 staining, is increased in the early days (24–72h) of culture and this has been attributed to growth promoting factors within the medium or release from systemic control upon removal from the patient [31]. Our data show that, using our defined medium and hydrogel support, cellular proliferation in normal breast explants remains unchanged throughout 7 days of culture. This, and the maintenance of PR expression, suggests that the low level of oestrogenic compounds in the phenol red containing medium are sufficient to maintain oestrogen receptor activation and signalling representative of that seen in the uncultured tissue. By day 7 there appears to be vacuolation of the myoepithelial cells which is typical of the normal breast as it enters the luteal phase of the menstrual cycle. This could suggest that the tissue is responding to progesterone, a component in the B27 additive, but this requires further investigation to confirm. The ability of a normal breast preclinical model to maintain unperturbed proliferation and hormone status is essential for investigating endocrine risk-reducing agents, such as tamoxifen, in vitro. Proliferation is a typical primary pathological end point in prevention trials and is robustly maintained in our model. Cell death is another common endpoint in preclinical drug testing and our established model conditions maintained close to 100% viability over 7 days, allowing any observed changes in viability after treatment to be measured. Our culture model is currently limited to 7 days as, although some of our explants were able to survive for longer periods, levels of ERα and PR began to fall. We hypothesise that changes in the hydrogel structure over time may be implicated in these changes [32] and passage of explants to a fresh hydrogel may overcome this issue, although this remains to be tested.

Our data show that an elastic modulus of 413.78 Pa was best suited to maintain normal breast characteristics during culture compared to stiffer hydrogels of 1472.11 Pa and softer hydrogels of 244.43 Pa. The variation seen between the different hydrogel elastic moduli show the importance of tight control during culture of the normal breast tissue in vitro with even small changes having profound effects. This model also offers the opportunity to experimentally adjust elastic modulus to mimic the changes resulting from higher mammographic density related to increased risk. This will allow the model to be used to answer questions about the impact of increased tissue density on the biology of the cellular and stromal components within these normal tissues. Currently our model is based upon a hydrogel enriched with arginylglycylaspartic acid (RGD), which is the most abundant integrin ligand found within the extracellular matrix [33]. Whilst this support has proven to be a good model, it will be interesting in the future to include other components such as collagen and hyaluronan as these molecules are known to be highly influential in the interaction between epithelial and stromal cells and the extracellular matrix [34, 35].

Our normal breast explant model not only shows excellent viability and maintenance of proliferation and hormone receptor expression over 7 days but also that explants remain responsive to hormone stimulation and antagonism. Typically, oestrogen receptor expression is highly variable in normal tissue and rarely remains stable during in vitro culture [19], making our model appealing for investigations into hormone receptor signalling. Response to the anti-oestrogen, fulvestrant, also suggests this model may be useful in testing the efficacy of endocrine and other drug classes in the preclinical setting.

It would also be of great interest to assess the cellular hierarchy within our explants, asking whether luminal progenitors/stem cells persist, and to investigate changes in gene expression in each subpopulation with and without treatment [36]. Assessment of the stromal compartment would also be interesting to assess changes or maintenance of fibroblast and adipocytes, for example. Immune cells persist in our explant culture over at least 7 days but have not yet been fully explored. Such cells play a vital role in tissue maintenance and in the response to certain therapies [37, 38]. Their persistence and role in the maintenance of tissue homeostasis is an active avenue of research and we will continue to refine the medium in this model in the hope of maintaining these cells for longer periods. Investigation of the short-term inhibition or stimulation of immune cells is feasible in the explant model and may shed light on their relative importance in determining the efficacy of preventative therapies.

We have established a foundation of characteristics, viability, proliferation and hormone responsiveness, that are crucial for a preclinical normal breast model to aid translational investigations. To build further confidence and proof of concept of our explant model we will need to assess gene expression changes in normal breast tissue pre- and post-explant culture. Preliminary unpublished data suggest that there is minimal change in gene expression through culture alone, but more patient samples are needed to confirm this. Ultimately, we will assess the ability of our explant model to truly recapitulate the clinical situation in vitro through the assessment of cellular characteristics and gene expression changes with pre- and post-tamoxifen treatment in culture and directly compare them to the gene expression changes in biopsies from patients before and during treatment with preventative tamoxifen (Biomarkers of Breast Cancer Prevention, BBCP. Funded by the Biomedical Research Centre (IS-BRC-1215–20,007)). If we can show that short-term responses in our explant model can mimic those in patients, we will have a useful tool for preclinical testing of novel agents that could be explored in the next generation of clinical prevention trials [36–38].

## Ethics approval and consent to participate

The authors declare no competing interests.

### Supplementary Information

**Additional file** **1**. Rheometry was performed, and elastic modulus calculated for low, moderate and high hydrogels (*n*=4). *** *P* <0.001.

## Data Availability

No datasets were generated or analysed during the current study.

## Abbreviations

BBCP: Biomarkers of Breast Cancer Prevention
BRC: Biomedical Research Centre
CM: Clevers’ medium
E2: 17β-Oestradiol
ECM: Extracellular matrix
ERα: Oestrogen receptor α
ExM: Explant medium
FCS: Foetal calf serum
MCRC: Manchester Cancer Research Centre
NR: Not recorded
PR: Progesterone receptor
RGD: Arginylglycylaspartic acid
TMA: Tissue microarray
