# Supplementary figures and images for: A novel preclinical model of the normal human breast
Source: J Mammary Gland Biol Neoplasia. 2024 May 2;29(1):9. doi: 10.1007/s10911-024-09562-4 (PMC11065935; doi:10.1007/s10911-024-09562-4)

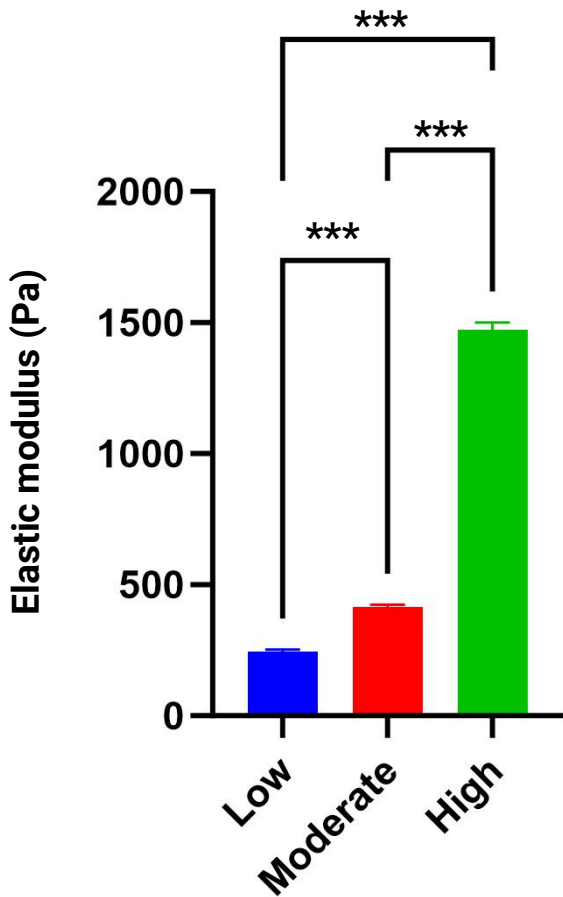

Supplement: Supplementary file 1 — Additional file 1. Rheometry was performed, and elastic modulus calculated for low, moderate and high hydrogels (n=4). *** P <0.001. [file 10911_2024_9562_MOESM1_ESM.pdf]
